# Oxidation Behavior of Nanocrystalline Alloys

**DOI:** 10.3390/ma17235842

**Published:** 2024-11-28

**Authors:** Yashaswini Karanth, Saurabh Sharma, Kris Darling, Haitham El Kadiri, Kiran Solanki

**Affiliations:** 1 School for Engineering of Matter, Transport, and Energy, Arizona State University, Tempe, AZ 85287, USA; 2 Army Research Directorate, DEVCOM Army Research Laboratory, Aberdeen Proving Ground, MD 21005, USA; 3 Michael W. Hall School of Mechanical Engineering, Mississippi State University, Mississippi State, MS 39762, USA

**Keywords:** oxidation, nanocrystalline, high temperature, alloys

## Abstract

Thermo-mechanically stabilized nanocrystalline (NC) alloys are increasingly valued for their enhanced mechanical strength and high-temperature stability, achieved through thermodynamic and kinetic stabilization methods. However, their fine-grained structure also increases susceptibility to internal oxidation due to higher atomic diffusivity associated with a greater volume fraction of grain boundaries (GBs). By incorporating solutes that form protective oxides, or the so-called thermally growing oxides (TGO), this vulnerability can be mitigated. The TGO scale acts as a diffusion barrier for oxygen that slows down the oxidation kinetics and prevents internal oxidation that impairs the structural integrity of the metal. This review examines advancements in oxidation-resistant NC alloys, focusing on the interplay between grain size and alloy chemistry. We explore how grain refinement influences diffusion coefficients, particularly the enhanced GB diffusion of Ni and Cr in Ni-Cr-based alloys, which improves oxidation resistance in NC variants like Ni-Cr and Cu-Cr compared to coarse-grained counterparts. We also analyze the role of third elements as oxygen scavengers and the impact of reactive elements such as Hf, Zr, and Y in NiAl alloys, which can slow down diffusion through early establishment of protective TGO layers and enhance oxidation resistance. The concomitant effect of grain size refinement, modifications in alloy stoichiometry, and enhanced atomic diffusion is shown to manifest via drastic reductions in oxidative mass gain, and visualization of the stable, protective oxide scales is delivered through characterization techniques such as TEM, SEM, and EDS. A brief overview is provided regarding stress effects and the role of induced stress in driving oxide scale spallation, which can negatively impact oxidation kinetics. Lastly, we propose future research directions aimed at developing micro-structurally stable NC alloys through multi-solute strategies and surface modification techniques, targeting robust materials for high-stress applications with improved oxidation resistance.

## 1. Introduction

Thermo-mechanically stable nanocrystalline (NC) alloys are a class of advanced materials that have recently gained traction as forerunners for high-temperature, structural applications owing to their numerous beneficial attributes. One key characteristic feature of this class of materials is their extremely fine crystallite sizes, in the range of tens of nanometers [1]. Previous studies have established the predominant role of grain boundaries (GBs) and interfaces in influencing a wide range of properties in these materials [2]. Those mechanical, electrical, thermodynamic, kinetic, and chemical properties that heavily rely upon grain size and GB phenomena [3,4,5,6,7] exhibit a marked improvement in NC materials compared to their conventional coarse-grained counterparts [8,9,10,11,12]. These properties include increased yield strength or hardness [13,14,15,16,17], enhanced plasticity [14,18,19], high coefficient of thermal expansion [20,21], and increased surface free energy [22,23,24,25]. Another salient trait exhibited specifically by NC alloys is the enhanced diffusivity within the alloy, which is also attributed to the increased density of the GB fraction [26,27,28].

Their mechanical and thermodynamic behavior makes these stabilized NC alloys a feature of extreme interest for use in high-temperature and advanced applications such as engine pistons, turbines, and liners, etc., where a combination of enhanced mechanical properties such as high-temperature micro-structural and thermal stability is crucial [5,29,30,31]. During the above-mentioned applications, these materials are more likely to be exposed to oxygen-rich environments, where they may be subjected to extremely corrosive conditions [11,13,32]. Therefore, besides being equipped with an arsenal of advantageous mechanical and thermodynamic properties, NC materials must also possess superior resistance to oxidation at elevated temperatures. Thus, it becomes necessary to study the high-temperature oxidation behavior of NC alloys to identify measures to improve their durability for their continued long-term use in these fields.

In the existing literature, researchers have described in detail the various techniques that can be employed to improve the oxidation resistance in commercially available alloys. Most commonly employed intrinsic measures include the addition of “oxygen-scavenging” elements in the form of third elements (such as Cr or Al) to promote selective oxidation in binary alloys, grain size refinement, alloying with trace amounts of reactive elements (such as Hf, Zr, and Y, etc.) [33,34,35], and extrinsic measures such as externally applied thin film coatings that are capable of developing thermally grown oxides (TGOs) and oxide-dispersion-strengthened (ODS) coatings, etc. [36,37,38,39,40]. Each of these measures offers a unique set of advantages and disadvantages to the end-goal application of such alloys. For example, while the addition of third-element oxygen scavengers such as chromium can result in the formation of a highly stable and protective chromia scale, the increased addition of Cr to an alloy can impart unprecedented brittleness and compromise the high-temperature mechanical stability of such an alloy. This consideration is particularly crucial for thermo-mechanically stable NC alloys, where significant solute engineering has been employed to achieve stability. Any added oxygen-active solutes must fulfill their intended function without disrupting the alloy’s primary stabilizing mechanism. Therefore, it is important to carefully consider the merits and demerits of all available processes for improving oxidation resistance during alloy design for high-temperature applications, keeping in mind the specific set of attributes required in the resultant alloy.

Moreover, it is important to emphasize that while studying the oxidation resistance behavior of any alloy, many governing criteria can affect the oxidation of NC alloys. These properties can then be classified into those intrinsic properties that inherently depend upon material behavior (such as grain size, self and inter-diffusion behavior, thermodynamic stability, creep behavior as it enters Stephenson formulation [41] for growth-stresses-assisted oxidation [42,43], and alloy composition) and extrinsic properties that are affected by environmental factors (such as synthesis techniques, resulting defect structures, and processing conditions). Though the NC structure can influence a wide variety of material properties, of particular relevance to this review are diffusion characteristics and thermodynamic parameters that can be affected by grain refinement through nanocrystallization, and how these can impact the oxidation resistance behavior in such alloys. The oxidation of any alloy can be divided into the following three stages:

1 An initial and transient stage wherein scales of the most thermodynamically stable oxide under given conditions are established;
2 The steady-state growth stage is wherein the established oxide scale grows in the normal direction to the alloy surface;
3 Breakaway oxidation, where the reservoir of the primarily oxidating element facilitating the TGO is depleted.

The formation of a “protective” oxide scale in the initial transient stage becomes crucial to imparting oxidation resistance to the underlying bulk alloy. The formation of such an exclusive, protective scale requires the selective oxidation of certain oxidizable elements present in the alloy, which can be described in five consequential steps:

1 Adsorption of oxygen gas to the alloy surface;
2 Nucleation of numerous individual protective oxide islands through rapid selective oxidation of the favored element;
3 Lateral growth of protective oxide nuclei to form a coalesced, continuous layer;
4 Thickening of a growing oxide layer and subsequent steady-state growth stage;
5 Spalling and exfoliation of the TGO layer amid breakaway oxidation or thermally cyclic effects accelerating depletion of the primary oxidating element.

This review presents results gathered from various researchers in the efforts aimed at improving oxidation resistance in alloys, specifically those revolving around promoting steps 2 and 3 as outlined above.

## 2. Influence of Nanocrystallization on Oxidation Behavior

### 2.1. Bulk vs. GB Diffusion in NC Alloys

**Figure 1:**
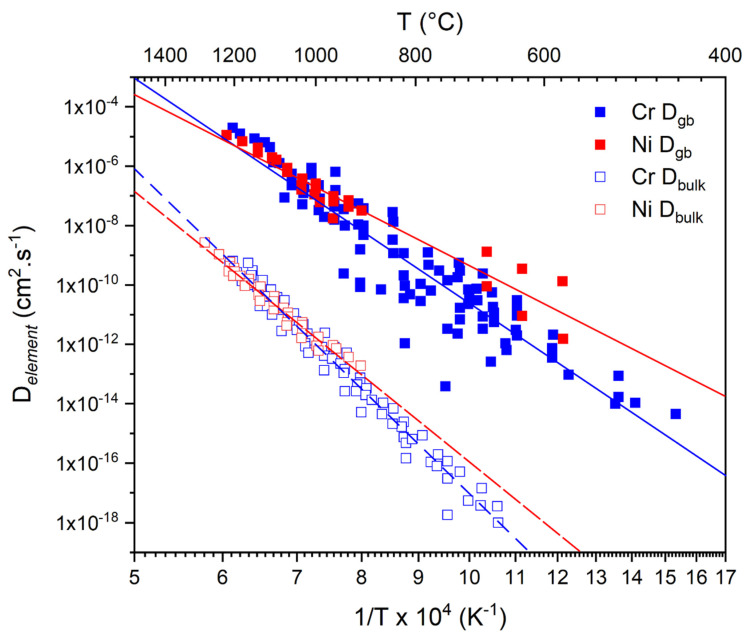
Tracer diffusion coefficients of Ni and Cr in pure Ni and various Ni-based superalloys of different compositions. Bulk and GB diffusion coefficients are marked with open and solid symbols, respectively. Blue symbols denote Cr and red symbols denote Ni diffusion coefficients. A full list of exact alloy composition and alloy specifications can be found in the reference [45].

It is widely established that the large volume fraction of GBs available in NC materials provides a path of higher diffusivity and thereby greatly enhances the GB diffusion coefficient in comparison to the bulk diffusion coefficient [44]. For instance, Huang et al. [45] have summarized the bulk vs. GB diffusion coefficients for Cr and Ni in pure Ni and Ni-based alloys, as shown in Figure 1 and conclusively establish the trend of increased diffusion along GBs. Similar trends have been observed for the diffusion of Al and Cr in varying NC–metal matrices and Cu, Ni, and Co-based NC–alloy systems (e.g., [45,46]).

The total diffusion coefficient of any species can be expressed as an effective value in terms of the bulk and GB diffusion, as given by Hart’s equation (Equation (1)).

(1) $$D_{eff}=\left(1-f\right)D_{bulk}+fD_{gb}$$

where *D_gb_* is the GB diffusion coefficient and *f* is the fraction of GBs. Therefore, by having a higher *D_gb_*, there is an increase in the effective diffusion coefficients. This increase in diffusion can lead to a faster migration of species during oxidation and lead to rapid oxide scale growth. In the presence of protective oxide-forming solutes like Cr or Al in the matrix, this faster oxide scale formation can slow down further oxidation of the alloy substrate. This contrasts with conventional coarse grain alloys that have a lower grain boundary fraction than NC materials and therefore lattice diffusion dominates the oxidation process. Further, nanocrystallization can also cause a reduction in the critical content of certain alloying elements required to confer superior oxidation resistance, which will be explored in Section 3. More advanced treatments of the diffusion coefficients of various species during alloy oxidation also take into consideration the interdiffusion coefficient, average diffusivity—which establishes a link between the mean stress gradient and different elemental fluxes—and the differential diffusivity arising due to the effect of the compositional gradient. A thorough review of such treatments and the consequent stress effects that can impact oxide scale spallation and severely influence oxidation behavior in NC alloys is provided later in Section 3.3.

### 2.2. Effect on Oxidative Kinetics

Understanding the effect of higher effective diffusion coefficients quantitatively during oxidation requires measurement of either oxide scale thickness or mass gain during experimentation. These procedures can give insights when comparing the oxidation resistance of various materials. In NC alloys, a refined micro-structure has proven to cause a significant reduction in the overall oxidative mass gain of numerous alloy systems that have protective oxide-forming solutes. Some notable alloy systems studied include NiCr and NiAl binary alloys, NiCrAl ternary alloys, various Ni-based superalloys, CuCr binary alloys, and CuNiCr ternary alloys to name a few.

**Figure 2:**
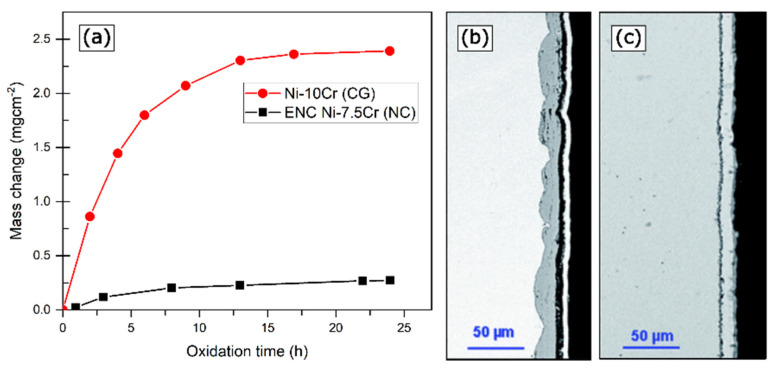
Oxidation kinetics in dry air at 900 °C of (**a**) electrodeposited NC-Ni-7.5wt.%Cr and CG-Ni-10wt.%Cr; cross-sectional SEM images of oxide scales developed on (**b**) CG-Ni10Cr and (**c**) ENC-Ni7.5Cr. Adapted from reference [46].

**Figure 3:**
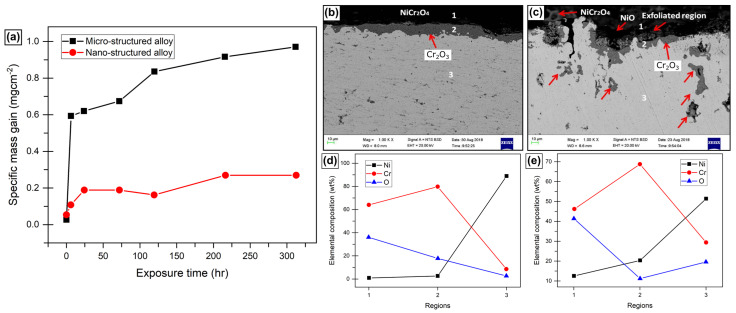
(**a**) Oxidation plot of specific mass gain of sintered micro-structured and nano-structured nickel-based alloys against time at 1100 °C. Cross-sectional SEM view images and corresponding EDS analyses after 312 h oxidation in air at indicated regions of (**b**,**d**) nano-structured Ni17Cr and (**c**,**e**) micro-structured Ni17Cr. Regions 1, 2, and 3 as marked on SEM maps (**b**,**c**) correspond to the sites of data acquisition in EDS scans as shown in (**d**,**e**). Adapted from reference [47].

Ni-based alloys are the major forerunners in improving oxidation resistance by refining grain size due to their extensive applications at elevated temperatures. In an early study, Peng systematically compared the oxidation behavior in a coarse-grained Ni-Cr binary alloy and an NC Ni metal matrix embedded with Cr nanoparticles, prepared via electrodeposition [46]. The oxidative mass gain curves for oxidation in dry air at 900 °C, carried out in a vertical tube furnace (refer to Figure 2a) and the oxide scale cross-sectional analysis (Figure 2b,c) showed a stark variation in the oxidation behavior of these two alloys [46]. CG-NiCr displayed a greater extent of oxidative attack, as seen by the higher mass change and thicker oxide layer. Similar behavior was observed in a separate study by Babalola et al. on the oxidation behavior of the binary Ni-17wt.%Cr alloy [47]. The isothermal oxidation examination (refer to Figure 3a) demonstrated that NC alloy has a higher oxidation resistance compared to its micro-structured counterpart. Further, the oxide scale analysis indicated a formation of chromium oxide scale in comparison to porosity and incomplete oxide scale in the micro-structured sample.

In both studies, the enhanced oxidation resistance associated with nanocrystallinity was attributed to two factors: the rapid diffusion of protective oxide-forming solutes, such as chromium (Cr) in the Ni-Cr binary system; and the increased number of oxidation sites due to a higher fraction of grain boundaries. For instance, in an NC Ni matrix, a uniform distribution of Cr nanoparticles would enable quicker formation of isolated Cr_2_O_3_ nuclei islands. These islands would grow laterally, speeding up the development of the chromia layer. This process requires the diffusion of Cr from the alloy to the oxidation front, and the higher diffusivity paths along grain boundaries in the NC Ni matrix would facilitate this diffusion. The faster diffusion of solute due to nanocrystallinity also contributed to the increased oxidation resistance in Ni-17wt.%Cr, as observed in Figure 3a. EDS spectra (refer to Figure 3d,e) collected at three different depths of the sample showed that the micron-sized Ni-17wt.%Cr contained NiCrO_4_ spinels on the surface, indicating that Ni diffusion to the oxidation front was not prevented during high-temperature oxidation. In contrast, the Ni content in the nano-structured Ni-17wt.%Cr remained low and negligible until the base metal depth was reached, where it stabilized at a concentration corresponding to the Ni content in the bulk alloy. This suggests the formation of a Cr_2_O_3_ layer during oxidation in the nanocrystalline alloy, which acted as a barrier to Ni diffusion, thereby improving oxidation resistance. On careful scrutiny of the oxidation kinetics in the nano-structured NiCr system, it is observed that the specific mass gain drops marginally before continuing to rise as oxidation progresses. Therefore, it can be hypothesized that this intermittent drop in mass gain can be attributed to oxide scale spallation and loss of oxide scales during the breakaway oxidation stage. Here, oxide scale spallation is attributed to the possible development of shear stresses within the developing oxide scale, due to the thermal gradient occurring between adjacent oxide layers as the scale develops and grows. This phenomenon, otherwise termed Stress-Aided Grain Boundary Oxidation (SAGBO) [48,49,50], has been studied widely and various studies have formulated mathematical treatments to estimate the stress developed in oxide scales during alloy oxidation, as well as to identify the breakaway stress at which oxide scale spallation is imminent in such alloys.

**Figure 4:**
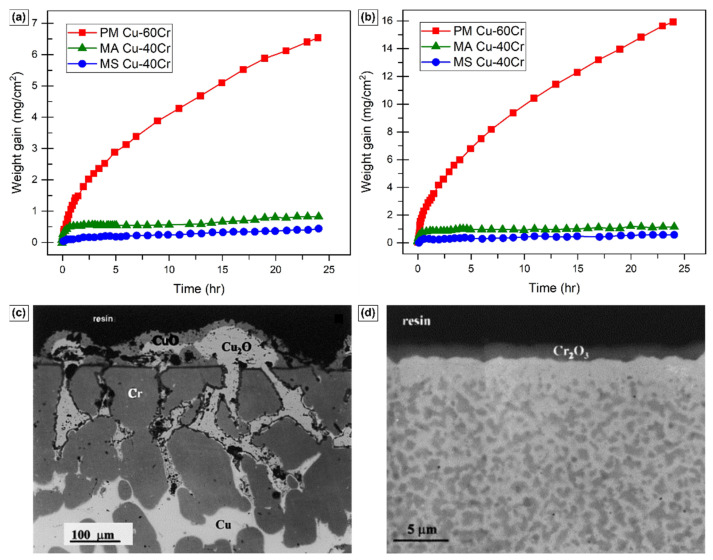
Isothermal oxidation behavior comparison in CG and NC prepared by various techniques at (**a**) 700 °C and (**b**) 800 °C. Oxide layer cross-section SEM images of samples prepared through (**c**) powder metallurgy (coarse-grained) and (**d**) magnetron sputtering (nano-grained) oxidized at 800 °C in 1 atm of pure O_2_ for 24 h. Adapted from reference [51].

Among Cu-based alloy systems, numerous researchers have extensively studied the addition of chromium (Cr) as a protective oxide-forming solute to enhance oxidation resistance [51,52]. Various processing methods, such as casting, powder metallurgy (PM), mechanical alloying (MA), equal channel angular pressing (ECAP), and magnetron sputtering (MS) have been employed to fabricate coarse-grained (CG) and nanocrystalline (NC) Cu-Cr alloys with different grain sizes. For instance, Fu et al. compared the oxidation behavior of coarse-grained Cu-60wt.%Cr prepared using PM with that of nano-grained Cu-40wt.%Cr fabricated using either MA or MS [51]. In the CG alloy, the grain size ranged from a few microns to 15 µm, while for MA alloy, exhibited a bimodal distribution with smaller grains in the range of 10–50 nm and larger ones around 200–300 nm. The average grain size after MS was between 5 nm and 10 nm. Figure 4 presents the oxidative weight gain comparisons in all three alloys at 700 °C and 800 °C for oxidation carried out in 1 atm of pure oxygen. It is apparent that as the grain size decreases from PM to MS, the weight gain also decreases, indicating a significant enhancement in oxidation resistance. Similar findings have been reported by Pan et al. while comparing cast and ECAP Cu-7at%Cr alloy in a similar temperature range [52]. Grain-refined Cu-7at%Cr alloy demonstrated better oxidation resistance than cast alloy.

The analysis of the oxide layer cross-section in these studies further emphasizes the significant influence of grain refinement on oxide formation. In CG micro-structures, intricate scales containing CuO, Cu_2_O, Cr_2_O_3_, and Cu_2_Cr_2_O_4_ have been observed contrasting with the nano-grained micro-structure produced via MS, where a fully developed Cr_2_O_3_ scale is visible, as illustrated in Figure 4c,d [51]. These results further support the statement that grain refinement can enhance the effective diffusion coefficient of ionic species, thereby fostering the selective oxidation of Cr and facilitating the development of a protecting chromia scale. Nevertheless, the formation of this chromia layer is contingent upon the concentration of Cr. In the instance of Cu-7at%Cr, owing to the low chromium concentration, zones rich in Cr_2_O_3_ were detected instead of a fully matured chromia layer, indicating the necessity of a critical Cr concentration for the formation of a protective scale [52].

In the case of both Cu-based and Ni-based alloy systems, nanocrystallization is shown to cause significant improvement in the oxidation resistance behavior of the alloy. Studies carried out in other alloy systems (such as Co-based [53] and Fe-based [54] alloys) also establish a similar trend. Altogether, grain refinement can promote the selective formation of a complete, uniform, and protective external oxide scale (such as chromia or alumina). In this manner, nanocrystallization plays a vital role in decreasing the extent of oxidative attack on the underlying bulk alloy, allowing for lengthened lifetimes, and there is a favored application of such alloys in high-temperature and harsh oxidative environments.

## 3. Influence of Alloy Chemistry on Oxidation Behavior

Alloy chemistry and composition play a crucial role in driving oxidation mechanisms, especially in ternary- and multiple-alloying-element NC alloys. The exact stoichiometry of certain alloying elements that promote the formation of protective external scales, the presence of trace amounts of certain reactive elements (such as Y, Zr, and Hf, etc.), and relative ratios of atomic sizes of alloying elements can all contribute to significant improvement of oxidation resistance.

### 3.1. Third Element Effect

**Figure 5:**
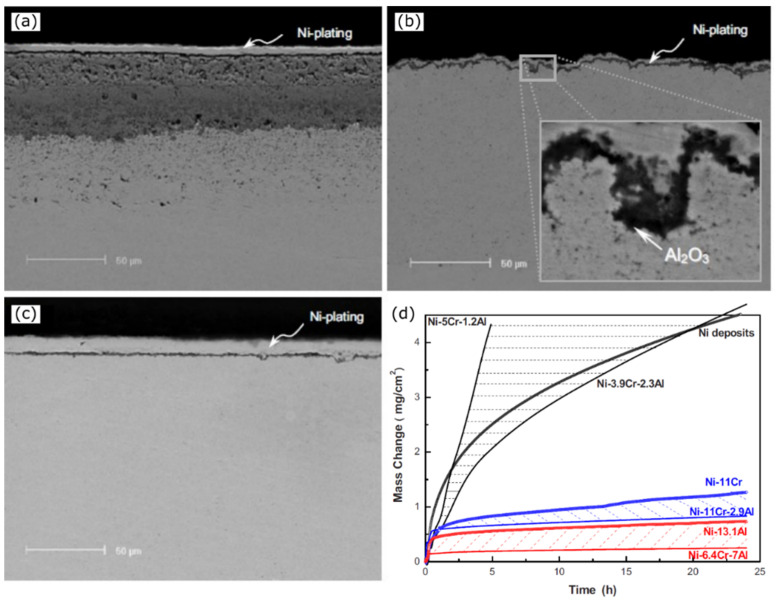
SEM images of cross-sectional features of developed oxide scales on Ni-xCr-yAl alloy oxidized in air at 900 °C for 24 h: (**a**) NiO forming Ni-3.9Cr-2.3Al; (**b**) Cr_2_O_3_ forming Ni-11Cr-2.9Al (inset shows the presence of localized islands of Al_2_O_3_); and (**c**) Al_2_O_3_ forming Ni-6.4Cr-7Al. Oxidation kinetics for the above samples are presented in (**d**), with reference oxidation mass gain curves of pure Ni deposits and typical NiCrAl nanocomposites. Images and data are taken from reference [55].

The third element approach is usually concerned with the addition of a third element to a binary alloy having intermediate reactivity compared to the base elements, which may act as an “oxygen scavenger” during the initial stages of oxidation. The oxygen scavengers decrease the oxygen solubility within the alloy, and favor the transition from internal oxidation of the most reactive element to its external oxidation, allowing for the selective and desired formation of external protective scales. Extremely detailed and comprehensive studies have been carried out by various researchers to understand this effect. Yang et al. verified the third element approach while studying the oxidation behavior of various compositions of the Ni-xCr-yAl ternary NC alloy system [55]. In this ternary alloy, Al acted as an oxygen scavenger compared to Cr or Ni due to its higher affinity toward oxygen and enhanced the selective oxidation and formation of the external oxide scale of alumina. Figure 5 provides a summary of the findings by Yang et al. in their study of various compositions within the Ni-xCr-yAl ternary NC alloy system [55]. Oxidation was carried out in a thermogravimetric analyzer in commercial dry air at 900 °C, with a 50 °C/min heating rate. The mass gain curves clearly show that oxidation resistance improves with increasing Al concentration, as seen in the comparison of Ni-5Cr-1.2Al, Ni-3.9Cr-2.3Al, Ni-11Cr-2.9Al, and Ni-6.4Cr-7Al. Additionally, the results indicate that the addition of Cr reduces the critical aluminum content required to form protective alumina scales while also enhancing the oxidation resistance of the system. This is evident when comparing Ni-13.1Al with Ni-6.4Cr-7Al, where the ternary alloy performs significantly better than the binary Ni-Al alloy.

**Figure 6:**
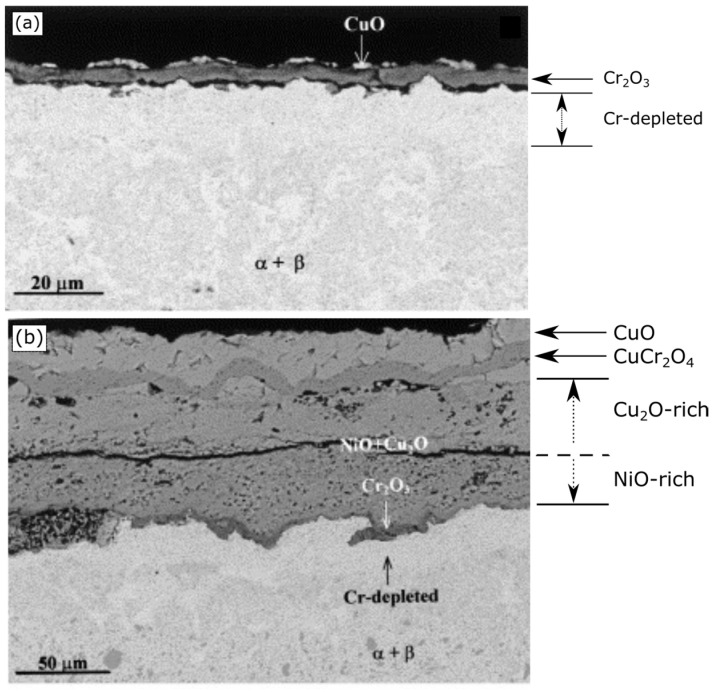
Oxide scale formation in MA (**a**) Cu-40Ni-20Cr and (**b**) Cu-20Ni-20Cr alloy after oxidation at 800 °C and 1 atm O_2_. Here, α (lighter phase) is Cu-rich phase, and is dilute in Ni and Cr. β (darker phase) is Cr-rich phase, as seen in SEM imaging of bulk alloy region. Taken from reference [56].

As mentioned above, the third element can act as an oxygen scavenger and enhance oxidation resistance. However, in immiscible systems like Cu-Cr, a third element can be selected based on its ability to improve the solubility of the protective oxide-forming solute, thereby facilitating the formation of an external oxide layer. For instance, Ni is usually added to binary Cu-Cr alloys to enhance the solubility of Cr and the formation of a protective oxide layer. Niu et al. [56] examined this phenomenon while studying the oxidation behavior of Cu-40wt.%Ni-20wt.%Cr and Cu-20wt.%Ni-20wt.%Cr. They observed that the alloy with a higher Ni content exhibited greater oxidation resistance. Oxide scale analysis, as illustrated in Figure 6, further revealed noticeable differences in the growth of the oxide layer in both alloys at 800 °C and 1 atm O_2_. With a higher Ni concentration, a continuous protective chromia scale can be observed, contrasting with the formation of multiple oxide layers when the alloy has a lower Ni concentration. This behavior was associated with the micro-structure where both compositions of the alloy exhibit a two-phase structure: a Cu-rich phase serving as the matrix; and Ni/Cr-rich phases distributed as particles within the matrix. However, the volume fraction of these phases varies depending on the concentration of Ni, with a higher proportion of the Cr-rich phase observed at higher Ni concentrations. Moreover, the size of the Cr-rich phases varies significantly. Niu et al. observed particles ranging from 1 to 4 µm at lower Ni concentrations, whereas at higher Ni concentrations, the size was much smaller, less than 1 µm [56]. A higher fraction of Cr-rich phases with smaller particle sizes has a better chance of nucleating and continuously forming a chromia scale, ultimately providing higher oxidation resistance. Huang et al. [57] further explored this behavior in electrodeposited Cu-Ni-Cr alloy, with Ni concentrations of either 30 wt.% or 50 wt.%, while maintaining the Cr concentration at 20 wt.%. They also observed a similar behavior where alloys with higher Ni concentration demonstrated lower weight gain in the Cu-Ni-Cr alloy.

Overall, the third element approach suggests that with proper selection of solutes, it is possible to design alloys that can selectively form certain oxide scales and repress the oxidation of other alloying elements within the alloy system, keeping in mind the rule of reactivity of these elements, which can be inferred from various thermodynamic tools such as Ellingham plots. For example, in NiCrAl alloys where high mechanical strength would be a favored outcome, the Al content can be tuned to minimize the critical content of Cr required to form a protective chromia scale. Cr addition (which would impart brittleness to the alloy) can then be controlled, allowing for the designed alloy to retain its desirable thermomechanical attributes, without compromising the oxidation resistance of this system.

### 3.2. Reactive Element Effect

**Figure 7:**
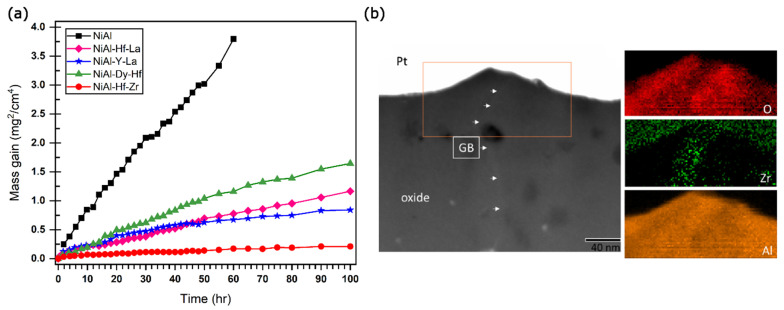
(**a**) Oxidation mass gain curves for various co-doped NiAl alloys. Data adapted from reference [60]. (**b**) HAADF STEM image of oxide ridge in scale developed on Zr-doped NiAl and corresponding EDS maps showing Zr-enrichment along the oxide GB. Taken from reference [61].

Reactive elements such as zirconium (Zr), hafnium (Hf), yttrium (Y), dysprosium (Dy), and lanthanum (La) are typically added to alumina- or chromium-forming alloys to enhance their oxidation resistance. When present in minor concentrations, these elements can reduce the oxide scale growth and improve scale adhesion. For instance, Hamadi et al. [58] demonstrated an improvement in oxide scale adhesion during the cyclic oxidation of NiAl doped with Zr compared with undoped NiAl alloy. Similar results have been presented in an early study by Jedlinski et al. [59] for a NiAl coating implanted with Y and cerium (Ce). However, an optimized concentration is crucial to fully utilize the benefits of reactive elements, as a higher concentration can lead to severe internal oxidation due to the strong oxide-forming ability of these elements, while a lower concentration may be insufficient to improve oxidation resistance. In recent years, a co-doping method has also been employed to mitigate the need for higher concentrations of reactive elements to improve oxidation resistance. This approach allows for enhanced oxidation protection while avoiding the issues associated with using high concentrations of reactive elements. For instance, Guo et al. investigated the effect of co-dopants like Hf-Dy, Hf-Zr, Hf-La, and Y-La on the oxidation behavior of a NiAl binary alloy [60]. Figure 7a shows the oxidation mass gain squared curves over 100 h of cyclic oxidation at 1200 °C. As evident, co-doping of the NiAl alloys profoundly decreases the oxidative mass gain, resulting in superior oxidation resistance exhibited by these alloys. Coprecipitation of reactive elements into phase-separate regions along the alloy GBs modified the diffusion behavior of various cationic species during alloy oxidation.

Further, a separate study by Boll et al. outlines the GB transport behavior of cationic species in the NiAl alloy system during high-temperature oxidation, the key result of which is presented in Figure 7b [61]. The conclusive evidence provided by the study strongly supports the above-mentioned hypothesis that GB diffusion and the modified diffusion behavior of cations profoundly affect oxidation behavior in the base alloy as compared to co-doped alloys. They studied the outward flux of diffusing Al ions as a function of the addition of trace amounts of reactive elements to the alloy. The oxidized samples were mechanically polished to remove the outer oxide scale layer and reoxidized to study outward Al diffusion flux, which manifested as the growth of small alumina ridges along the GBs on the oxide surface. By quantifying the area of these ridges, the outward flux was calculated using concentration-dependent diffusion laws. Figure 7b shows the HAADF STEM image of a GB and the accompanying growth of external alumina ridge in Zr-doped NiAl. Accompanying EDS elemental maps show the enrichment of Zr along the oxide GBs, extending into the outward-growing alumina ridge.

Through the concomitant effect of GB diffusion enhancement through nanocrystallization and the effect of the addition of reactive elements, it is possible to promote the formation of a complete and protective external oxide scale. The oxidation kinetics in nanocrystalline alloys are governed by various diffusional fluxes, namely the outward diffusion of protective scale forming cations (such as Al, Cr, and Si) and the inward diffusion of oxygen. To provide the underlying alloy with superior oxidation resistance, it is important to facilitate the rapid movement of the former and hinder the latter to the greatest extent possible. Once an oxidation reaction has been initiated, the smaller scale-forming cations on the surface can form nucleating islands of the protective oxide (such as alumina or chromia), which grow with continued slow outward diffusion of the cations. The larger ionic size of the reactive elements (such as Y, Hr, and Hf, etc.) retards their motion through the alloy, and also hinders the continued inward movement of oxygen through the alloy, thereby preventing internal oxidation and metal consumption within the alloy. Multiple studies are currently underway to provide deeper and more comprehensive substantiation to back the proposed role of reactive element addition in improving oxidation resistance through diffusion-controlled kinetics. Overall, the above discussion highlights the crucial role of alloy chemistry in controlling and tuning the oxidation response of NC alloys. The interplay of nanocrystallization-driven grain refinement and alloy stoichiometry provides interesting perspectives on the driving mechanisms in play during oxidation reactions in such alloys.

### 3.3. Effect of Growth Stresses

An important aspect of alloy oxidation is the stress-induced cracking of oxide films. This is clearly demonstrated by the excessive void formation observed in various alloy systems, including the NiCr system (Figure 3c), the NiCrAl system (Figure 5a), and the CuNiCr system (Figure 6b). Additionally, evidence of oxide scale spallation can be seen in Figure 3a. Numerous studies have investigated the tendency of thermally grown oxide scales to crack under the development of tensile stresses or to undergo spallation due to compressive stresses, which can result from phenomena such as buckling or wedging [62,63,64]. For instance, it is well-known that the selective oxidation of alloys induces growth stresses in the metallic substrate (and oxide scales) that can exceed 1 GPa [42,43,65].

Stress gradients develop in the substrate as a result of interdiffusion and dislocation climb-mediated vacancy replenishment at the metal–oxide interface, which recedes as metal mass loss [43]. To model this, Suo et al. [43] developed governing equations for density field and stress field developments in the metallic substrate. They comprise two driving forces for diffusion—the concentration gradient, which scales with the interdiffusion coefficient, and the mean growth stress gradients (hydrostatic pressure, $\sigma_{m}$), which scales with the interdiffusion coefficient (D) and differential diffusivity (Δ = D_A_ − D_B_):

(2) $$\frac{\partial c}{\partial t}=\frac{\partial }{\partial z}\left[D\frac{\partial c}{\partial z}-\frac{\Omega c\left(1-c\right)\Delta }{\phi kT}\frac{\partial \sigma_{m}}{\partial z}\right]$$

(3) $$D_{inter}=\left(1-c\right)D_{A}+cD_{B}$$

where $\frac{\partial c}{\partial t}$ is the concentration gradient; *D* is the interdiffusion coefficient (*D_inter_*); *z* represents an imaginary plane normal to the oxide surface across, from which the net flux of diffusing atoms is calculated; Ω is the alloy volume per atom (assumed constant); ϕ is Darken’s thermodynamic factor relating mobility and diffusivity; Δ is the differential diffusivity; and $\sigma_{m}$ is the mean stress [43].

However, stress relaxation by creep requires a second differential equation to update both the composition and stress fields:

(4) $$\frac{\partial c}{\partial t}=\frac{\partial }{\partial z}\left[D\frac{\partial c}{\partial z}-c\left(1-c\right)∆\frac{\partial \sigma }{\partial z}\right]$$

The concentration-gradient-dependent diffusional flux (*J*) can be related to a diffusion-induced strain rate value through the following equation:

(5) $$d_{x}^{D}=-\frac{\Omega \partial J}{3\partial z}$$

The expression for the transverse strain rate under biaxial stress state for both tension and compression conditions can then be expressed as follows:

(6) $$\sigma {\left|\sigma \right|}^{n-1}=\frac{\partial }{\partial z}\left[-∆\frac{\partial c}{\partial z}+D\frac{-\partial \sigma }{\partial z}\right]$$

where *n* is the stress exponent (presumed to be in the range 3–5), and where now stress affects the process via the average diffusivity.

(7) $$D_{aver}=\left(1-c\right)D_{B}+cD_{A}$$

This model can describe two processes generating stresses during oxidation: (1) cationic depletion of alloy element A near the alloy–oxide interface, causing divergence in the flux rates of alloy elements A and B; and (2) consumption of metallic ions at the alloy surface and their subsequent emission into the metal substrate as oxidation progresses. The model also includes boundary conditions that relate generated strain rates to diffusional flux within the alloy interior, forming the basis for understanding creep effects during oxidation.

Further research based on this model suggests that void formation and growth during oxidation are driven by tensile stresses developed both in the bulk and at the metal–oxide interface [42]. The simultaneous generation of voids and recession of the interface is mitigated by stress generation. A more detailed model was developed to explain the void formation, growth, and the subsequent influence on oxide scale spallation once voids propagate along the oxide–metal interface [42]. This model accounts for the curvature of voids, composition gradients, diffusional fluxes, and growth stresses; however, it does not consider the impact of grain size refinement, which can affect creep and dislocation behavior in NC alloys. As a result, the stress effects during oxidation in NC alloys differ significantly from those in conventional coarse-grained alloys. In particular, the distinct creep properties of NC alloys may alter mean stress gradients, influencing overall oxidation kinetics. Also, these effects are further complicated by the sign of the stress, which depends on the oxidation regime (cationic or anionic) and the stage of oxidation (early vs. steady-state). Additionally, reactive elements are known to enhance the anionic regime in alumina-forming alloys by stabilizing the HCP variant of the oxide phase, promoting faster growth, lower adherence, and greater susceptibility to exfoliation. The relationship between this mechanism and grain size remains insufficiently understood, highlighting a critical knowledge gap that warrants further detailed investigation.

## 4. Perspective

### 4.1. Micro-Structurally Stable Bulk NC Alloys

**Figure 8:**
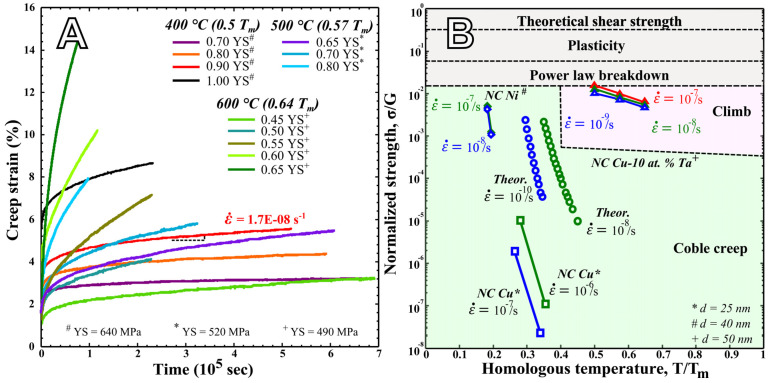
(**A**,**B**) Compression creep behavior of NC Cu-10at%Ta alloy [13].

Nanocrystalline alloys demonstrate enhanced oxidation resistance by consistently showing a reduction in oxidative mass gain compared to conventional alloys, attributed to their rapid formation of a protective oxide scale. This process is chiefly regulated by the more rapid formation of the TGO layers as a higher density of GBs offers fast diffusion pathways in the transient regimes toward the metal surface. Consequently, an increase in grain size during oxidation can result in a decrease in the effective diffusion coefficients, potentially leading to diminished oxidation resistance. To demonstrate the magnitude of grain growth, an eightfold increase in Cu-Ni grain size has been observed in Cu-rich Cu-Ni-Cr alloys after one hour of oxidation at 800 °C [57]. Similar findings have been documented for pure metals such as Ni [66] and Cu [67], as well as for numerous other alloy compositions. This emphasizes the significance of investigating micro-structurally stable NC alloys to minimize changes in diffusion coefficients during oxidation. By attaining this stability, the third element approach previously discussed can be effectively utilized. This involves incorporating elements such as Cr or Al into the alloy system, which then react to form protective oxides, thereby enhancing the oxidation resistance of these alloys. Over the past decade, considerable research efforts have been dedicated to attaining micro-structurally stable bulk NC alloys. One of the most promising strategies involves alloying metals with carefully selected solutes. These solutes serve to suppress grain growth, either by reducing GB free energy or by minimizing GB mobility, based on the principles of thermodynamics and kinetics, respectively [11,68,69,70]. Binary systems such as Fe-Zr [71], Ni-Y [72], and Cu-Ta [73,74,75,76,77,78,79,80,81,82,83,84] have demonstrated notable thermal stability at very high temperatures through the application of these approaches. Furthermore, these systems, particularly NC Cu-Ta with a grain size of <100 nm, have demonstrated exceptional thermo-mechanical behavior [13], refer to Figure 8A,B. This alloy system has demonstrated creep rates in the order of 10^−8^ s^−1^, which is 6–8 orders of magnitude lower than previously reported creep rates in NC metals. This exceptional behavior was linked to the presence of Ta nanoclusters in the micro-structure which were effectively inhibiting the grain coarsening during creep. Expanding the application of this system to understand oxidation can help fabricate high-strength alloys with superior oxidation resistance.

A further improvement in oxidation can also be suggested through suitable solute addition (or third element approach), such as Cr in the presence of Ta in NC Cu-Ta alloys. The choice of the third element depends on the criteria that the solute should be immiscible with the Cu matrix. Cr is immiscible with Cu; hence, it will allow independent exploitation of Cr particles to improve oxidation. However, the micro-structural stability of these NC alloys heavily relies on the distribution of solutes, and adding an extra solute can alter this distribution, ultimately affecting the thermal stability of these alloys. For example, in the case of Cu-Ta alloys, the mechanical strength is primarily contributed to by Ta nanoclusters and their interaction with dislocations [13]. Any changes in the formation of Ta nanoclusters due to introducing a third element, such as Cr, can lead to alterations in the alloy’s grain size, which would affect performance. Therefore, systematic investigations based on solute concentrations, phase formations, and environmental conditions are necessary. Nevertheless, these NC alloys have the potential to become the next-generation materials for structural and high-heat-flux applications across industries ranging from transportation to the energy sector.

### 4.2. Surface Mechanical Attrition Treatment (SMAT)

SMAT is a process that can be used to create a nano-structured surface on bulk materials by inducing plastic deformation. Unlike bulk NC alloys, the alloys generated through this technique feature refined grains exclusively on the top surface layer of the material [85,86,87]. This layer can extend to a thickness of several tens of microns. Due to grain refinement, these SMATed surface offers significant improvement in mechanical properties such as an increase in hardness, higher yield strength, wear resistance, and fatigue improvement [85,88]. In the context of oxidation, nanocrystallization would enhance the diffusion of ionic species within the grain-refined surface layer and initiate a rapid development of the oxide scale. Initially, one might perceive an increase in the oxidation rate; however, as the protective oxide scale forms, the rate will decrease, and the SMATed alloys may even outperform conventional ones. This phenomenon has been observed during the oxidation of P91 steel where SMATed samples outperformed conventional samples after extended hours of oxidation [89]. This design strategy has also been verified in other alloy systems like 304L stainless steel [90]. Applying this design approach to other alloy systems, particularly ternary alloys where Cr and Al serve as third elements, shows potential for creating materials with improved oxidation resistance. This is due to the rapid formation of Cr or Al oxides on the alloy surface. Additionally, these alloys can exhibit superior mechanical properties attributed to grain refinement. Furthermore, the flexibility and affordability of this approach would facilitate its seamless integration into a wide array of existing manufacturing processes.

## 5. Summary

In summary, this review examines the oxidation behavior of NC alloys as compared to conventional coarse-grained alloys. The refinement of grain size through nanocrystallization, coupled with the strategic adjustment of alloy composition—particularly the inclusion of oxygen scavengers—improves the formation of protective oxide scales. These modifications facilitate rapid grain-boundary diffusion, which promotes the selective oxidation of alloy components and reduces the oxidation of base metal elements during the initial stages of oxidation. As a result, NC alloys exhibit faster formation of stable oxide layers and transition to the steady-state oxidation phase more quickly, as evidenced by their parabolic mass gain curves. This enhanced oxidation resistance occurs without compromising other critical material properties, making NC alloys attractive for high-temperature and harsh oxidative environments. This review also explores how micro-structural stability, alloy stoichiometry, and the role of third elements contribute to the overall oxidation performance of NC alloys, positioning them as a promising material class for applications requiring both high durability and oxidation resistance.
